# A Compact Orthosis Compliance Monitoring Device Using Pressure Sensors and Accelerometers: Design and Proof-of-Concept Testing

**DOI:** 10.3390/s25051352

**Published:** 2025-02-22

**Authors:** Devi Baruni Devanand, Matthew D. Gardiner, Angela E. Kedgley

**Affiliations:** 1 Department of Bioengineering, Imperial College London, London SW7 2AZ, UK; d.devanand19@imperial.ac.uk; 2 Kennedy Institute of Rheumatology, Nuffield Department of Orthopaedics, Rheumatology and Musculoskeletal Sciences (NDORMS), University of Oxford, Oxford OX3 7FY, UK; matthew.gardiner@kennedy.ox.ac.uk; 3 Department of Plastic Surgery, Wexham Park Hospital, Frimley Health NHS Foundation Trust, Slough SL2 4HL, UK

**Keywords:** orthotics, design, hand, feasibility, compliance, adherence, pressure, sensor, accelerometer, wear time

## Abstract

Monitoring orthosis compliance using patient diaries is subjective, as patients can overestimate their levels of device use. An objective way to monitor compliance is required because if an orthotic prescription is not followed, the orthosis will not work as intended. This study aimed to develop and validate a device that monitors orthosis compliance objectively using pressure and acceleration. Fifteen participants were recruited to test the device’s ability to estimate wear time during the performance of several grip patterns and whilst completing selected activities of daily living. Sensor threshold values were used to discern whether users were wearing their orthosis or not. No differences between pressure sensor and accelerometer-based wear time estimations were found. The device’s pressure-based wear time estimations were found to have a specificity of 92.7 ± 16.4% and sensitivity of 74.0 ± 41.3%, whilst accelerometer-based wear time estimates had a specificity of 66.1 ± 34.7% and sensitivity of 86.2 ± 8.0%. This study successfully demonstrated the feasibility of monitoring hand orthosis compliance using pressure or acceleration. This device has the potential to provide insight into the effectiveness of both existing and novel orthotics, benefitting both clinical practice and research.

## 1. Introduction

With 1.2 million orthotics users in the UK [1], orthoses are commonly used in the treatment of a variety of conditions, such as osteoarthritis [2], rheumatoid arthritis [3], fractures [4], stroke-induced deficits [5], cerebral palsy [6], and chronic spinal conditions [7]. Additionally, they have been used for injury prevention [8]. Whether it is to control joint alignment, correct deformity, or prevent injury, orthoses are frequently deployed as a non-invasive and inexpensive treatment option. Upper limb orthoses can be static, or dynamic in one plane of motion, but they share the common goal of facilitating successful treatment by maintaining alignment, assisting grasping, preventing contractures, protecting joints, increasing functionality, increasing range of motion, correcting deformity, reducing pain, and/or immobilizing joints [9,10].

Compliance, or adherence, to treatment is defined as the degree to which a person’s behaviour agrees with recommendations from a health care provider [11]. Whilst orthoses are prescribed to relieve symptoms of conditions, the evidence highlighting their effectiveness is underdeveloped [12,13]. In research evaluating the efficacy of orthoses, an objective monitoring technique is required to ensure participants wear their orthoses as instructed because without optimal wear, there is no way of knowing the true impact of the orthotic treatment being investigated [14]. Furthermore, monitoring compliance is vital to understand how the orthotic treatment affects the patient’s wellbeing [15]. Clinically, information regarding compliance is immensely valuable when determining whether poor compliance to a treatment plan may play a role in the apparent failure of a device to aid in a patient’s recovery [16].

Patients stop complying with orthotic treatment for various reasons. For example, in one study, discomfort and disruption to the completion of activities of daily living led to patients with Duchenne muscular dystrophy discontinuing the use of their hand orthoses as prescribed [17]. Further reasons for discomfort included an ill-fitting orthosis, skin irritation, and sleep disturbance [16,17,18]. Discomfort arising from psychological factors such as low self-esteem, feeling different from peers, and the perception of a social stigma associated with wearing an orthotic device have also impacted compliance with orthotic treatment [19]. Moreover, the length of the treatment provided, the patient’s attitude towards the treatment, and their confidence in the clinical team providing it have all been shown to influence compliance [19].

Common techniques to monitor patient compliance with orthoses include patient diaries [20], questionnaires [21], and interviews [22]. However, due to their subjective nature, these methods are prone to social desirability bias as patients tend to overestimate their levels of compliance [16,23] and over-report ‘good behaviour’ [24]. Additionally, when compliance is assessed weeks or months after prescription, these methods also become vulnerable to recall bias, as exact wear time becomes harder to recollect [17]. It should be noted that a patient’s behaviour can also be affected when they know that their compliance to the treatment is being monitored [25]. Consequently, to overcome the human factors that compromise the accuracy of compliance measurements, an objective orthosis compliance monitoring tool is of great need.

Outcomes from a systematic review examining existing objective compliance monitoring techniques for orthoses revealed that the majority of studies employed orthoses for the lower limb, which highlighted the need for further development of objective monitoring techniques for the upper limb [26]. Additionally, all studies that did perform compliance monitoring of upper limb orthoses used temperature sensors [23,27,28,29,30], except one, which added pressure sensors [30]. This further established that there is a lack of evidence demonstrating the feasibility of a range of objective compliance monitoring techniques for the upper limbs.

There are many upper limb orthoses, both custom and commercially made, available today. Whilst upper limb orthoses are used in the treatment of a multitude of conditions, osteoarthritis was the condition of interest. Osteoarthritis of the hand has been described as a heterogenous disorder, often affecting multiple joints [31], including the trapeziometacarpal, metacarpophalangeal and interphalangeal joints [32]. Osteoarthritis at the trapeziometacarpal joint is strongly associated with age, affecting 33% of post-menopausal women [33] and 91% of those over the age of 80 years [34]. Equally important to note is that the trapeziometacarpal joint experiences large compression forces, meaning that mechanical load is also a major risk factor for the development of osteoarthritis [35,36]. Consequently, with an estimated lifetime risk of 39.8% [37], hand osteoarthritis burdens many in society.

The use of orthotics in the conservative care of the trapeziometacarpal joint pain is well established [38] and is recommended by guidelines for the management of hand osteoarthritis [39,40,41]. Orthotic devices prescribed to patients with osteoarthritis are mainly recommended for symptom relief [39], yet evidence highlighting the effectiveness of orthoses for trapeziometacarpal osteoarthritis is underdeveloped, with no evidence for the superiority of one thumb orthosis over another [42]. Whilst hand therapists prefer to prescribe custom-made orthoses to patients with hand osteoarthritis [43], the Push^®^ ortho Thumb Brace CMC (Nea Company, Maastricht, The Netherlands) is an off-the-shelf alternative that has been reported by users to provide more freedom of movement [44]. The Push^®^ orthosis supports the carpometacarpal (CMC) joint and does not affect the mobility of the other joints in the hand and wrist [45].

This study aimed to develop and validate a device that monitors orthosis compliance objectively using pressure sensors and accelerometers. The Push^®^ ortho Thumb Brace CMC was chosen in this study to test the orthosis monitoring device as it is available off the shelf and used in the treatment of base of thumb osteoarthritis. Furthermore, the Push^®^ ortho Thumb Brace is a rigid orthosis, which is beneficial when testing the compliance monitoring device, as it allows sensors to be attached securely.

## 2. Materials and Methods

### 2.1. Compliance Monitoring Device

**Figure 1:**
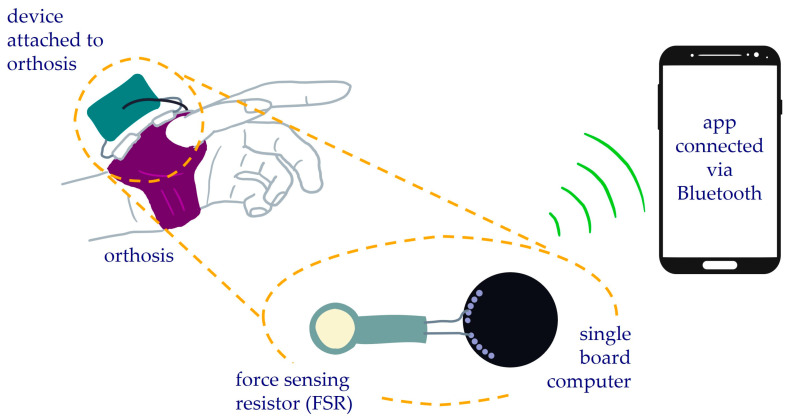
Orthosis compliance monitoring device setup comprising of a force sensing resistor attached to a miniature single board computer with an in-built 3-axis accelerometer, connected to a mobile phone application via Bluetooth.

In contrast to many previous monitoring systems this device was developed to be a self-dependent system that is compact, portable, and can visualise, obtain, and store data wirelessly. The custom-made orthosis compliance monitoring device was created comprising of a force sensing resistor (FSR, FSR07CE, Ohmite Manufacturing Company, Warrenville, IL, USA) wired to a miniature single board computer with an in-built three-axis accelerometer (MetaMotionC, Mbientlab Inc., San Francisco, CA, USA), connected to a mobile phone application via Bluetooth (Figure 1). The mobile phone application was developed using Java on Android Studio (version 2021.2.1, Google LLC, Mountain View, CA, USA) and tested on a Samsung Galaxy S9 phone (Samsung Group, Seoul, South Korea). The MetaWear Android API (Mbientlab Inc., San Francisco, CA, USA) was used to communicate with the MetaMotionC (MMC) board when connecting to Bluetooth and to save data streamed, at a sample rate of 1 Hz, from the FSR and accelerometer.

### 2.2. Electronics

**Figure 2:**
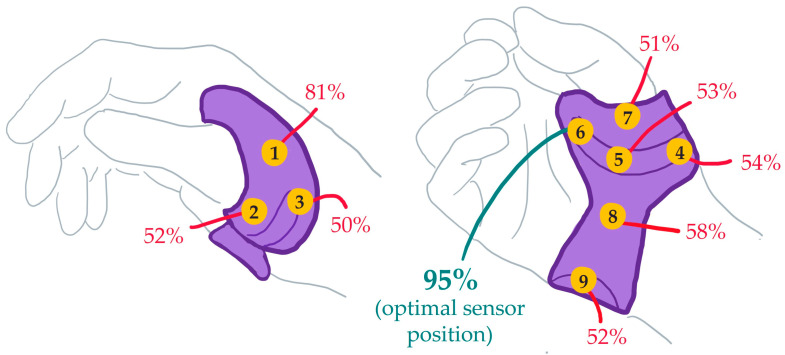
The force sensing resistor’s accuracy, calculated as the percentage agreement between actual and estimated wear times obtained while the orthosis was donned and doffed ten times, for various positions within a Push^®^ orthosis.

**Figure 3:**
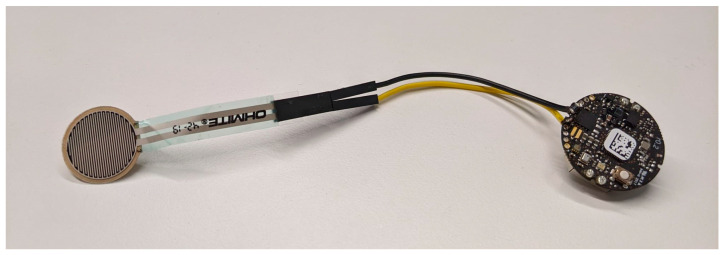
The orthosis compliance monitoring device after electronics were compacted.

**Figure 4:**
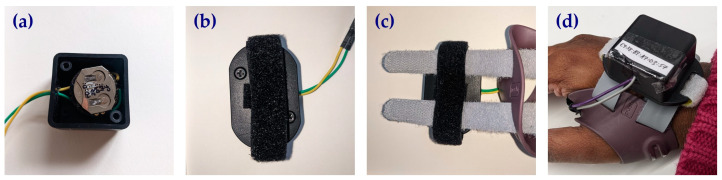
The final prototype of the orthosis compliance monitoring device fitted in an off-the-shelf enclosure. (**a**) Electronic components in enclosure. (**b**) Device enclosed. (**c**) Device attached to the straps of the orthosis. (**d**) Orthosis instrumented with compliance monitoring device worn by user.

A single board computer was employed to obtain data, determine the status of orthosis wear, and output this result securely. The CR2032 coin-cell battery powered MMC board was chosen, as it allowed the integration of peripheral pressure sensors whilst also having a built-in accelerometer and the option of incorporating a built-in temperature sensor. Furthermore, it also has the capability to connect via Bluetooth to a smartphone app which can sync and download data obtained from the sensors in real-time. The optimal position for the FSR within the orthosis was selected after nine suitable positions were chosen using landmarks on the orthosis and then tested. Wear time results were obtained from the FSR whilst a user donned and doffed the orthosis 10 times for each position. Wear time was estimated using a threshold value obtained through preliminary testing after which the FSR’s accuracy at each position was calculated as the percentage agreement between actual and estimated wear time (Figure 2). The final prototype consisted of the FSR attached to the MMC board (Figure 3), fitted in an off-the-shelf plastic enclosure with a VELCRO^®^ handle fixed to enable the device to be attached to the straps of the orthosis (Figure 4). The MMC board, containing the accelerometer, was positioned in parallel to the base of the device’s enclosure and facing upwards in the dorsal direction of the hand when the orthosis was worn.

### 2.3. Participants

Ethical approval for this study was obtained from the Imperial College Research Ethics Committee (ICREC reference: 21IC7308). To participate, volunteers were included if they were aged between 18 and 80 years and had no history of hand, wrist, or arm injury that affected their ability to perform activities of daily living (ADLs) within the 12 months prior to data collection. Information regarding the participant’s age, gender, hand dominance, and hand circumference were collected and documented upon their arrival at the laboratory.

### 2.4. Experimental Protocol

**Figure 5:**
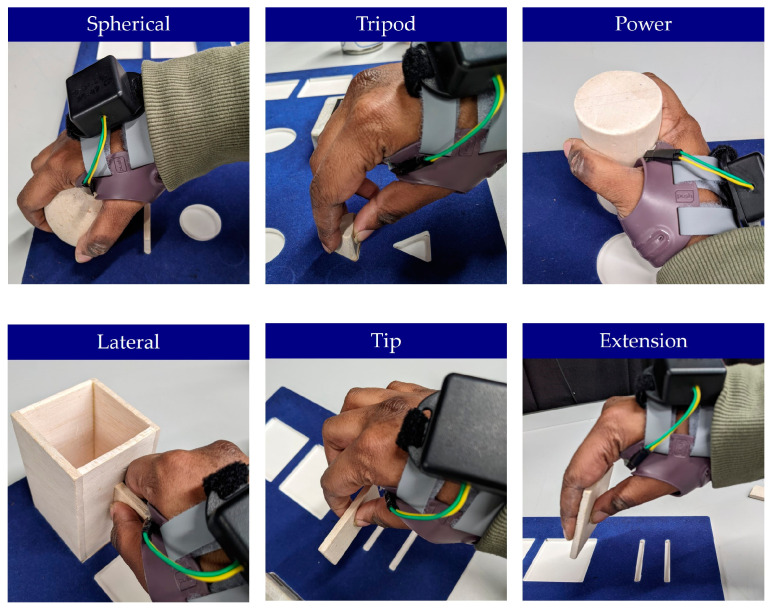
The first part of the Southampton Hand Assessment Procedure (SHAP). Participants were required to lift six abstract objects from one slot to another using a different grip pattern for each. This was completed using both light and heavy abstract objects.

**Figure 6:**
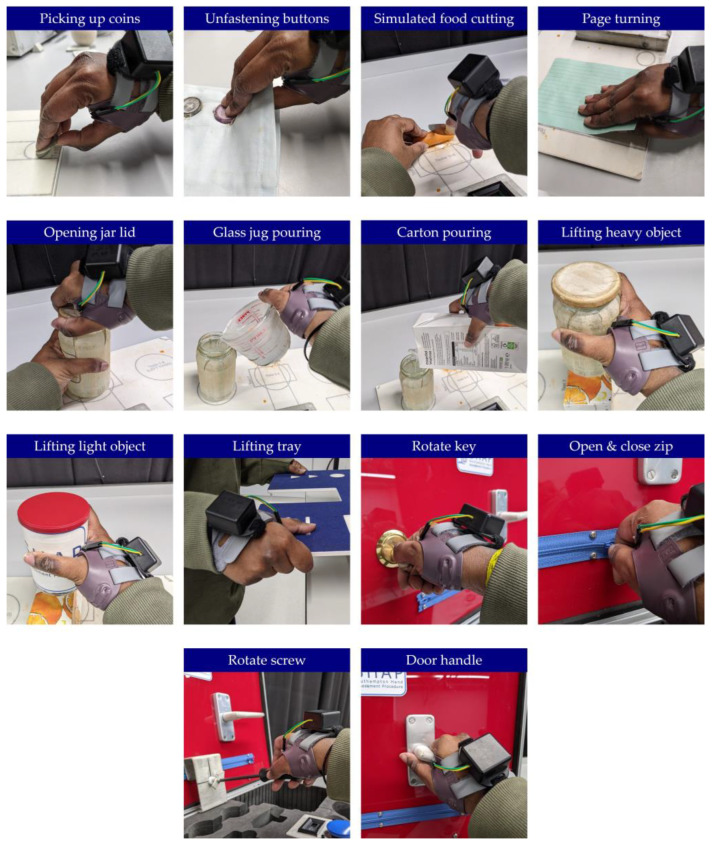
The second part of the Southampton Hand Assessment Procedure (SHAP). Participants were asked to perform 14 different activities of daily living (ADLs) whilst wearing the orthosis instrumented with the compliance monitoring device.

Participants were provided with an instrumented orthosis that best fit their dominant hand’s circumference (left or right in size small, medium, or large), as recommended by the Push^®^ ortho Thumb Brace CMC guidance. Following this, participants were given instructions on how to wear the instrumented orthosis and were asked to don and doff it. The times that the orthosis was worn and taken off were recorded both by timestamping on a computer and through an app collecting the data. To investigate the device’s accuracy during repeated orthosis use, the first part of the laboratory testing protocol consisted of the participant donning and doffing the orthosis ten times. Following this, the second part of the study focused on analysing the device’s accuracy during typical daily activities an orthosis user may complete. Thus, whilst wearing the orthosis, participants were asked to perform the Southampton Hand Assessment Procedure (SHAP), which consists of interaction with six abstract objects (Figure 5) and performance of fourteen ADLs that require grip (Figure 6) [46]. For each activity participants timed themselves using the SHAP timer by pressing start before they commenced the activity and stop after they completed the activity. To log wear time objectively, the investigator logged timestamps on a spreadsheet to mark when users donned and doffed the instrumented orthosis and when they started and stopped each activity of the SHAP. To minimise and avoid the effects of fatigue, participants were given one minute to rest between each of the activities. Once complete, participants were asked to fill out a short questionnaire regarding the comfort of the instrumented splint. The survey consisted of questions asking participants, on a scale of 1 to 10, how comfortable it was to wear the orthosis instrumented with the device, how comfortable they believed it would be to wear the instrumented splint for a longer period, and how easy it was to remove and replace the battery. The survey also offered participants the opportunity to provide feedback regarding possible improvements. Each session lasted no longer than 1.5 h.

### 2.5. Data Analysis

Data, collected by the device using the FSR and accelerometer, were downloaded from the mobile phone, and wear time was determined using threshold values obtained through preliminary testing for both the FSR and accelerometer. Threshold values were chosen to determine the cut-off point below which data saved from the sensors would be classified as non-wear. To minimise errors arising from sensor-to-sensor variability in the accuracy of pressure measurements, wear time estimations were calculated by thresholding the raw values obtained from the FSR and consequently, a threshold analogue-to-digital converter (ADC) value of 10 counts, corresponding to no pressure applied (0 Pa), was chosen for the FSR. A threshold value of 0.989 g was used for the accelerometer, found by averaging the vector magnitude obtained when the device was doffed and at rest for a minute. To monitor compliance, data above the threshold values meant that the orthosis was worn and were given the value of 1 and data below the threshold value corresponded to non-wear times and were given the value of 0. The binary values obtained from each sensor were compared to the actual wear time logged whilst donning and doffing.

**Figure 7:**
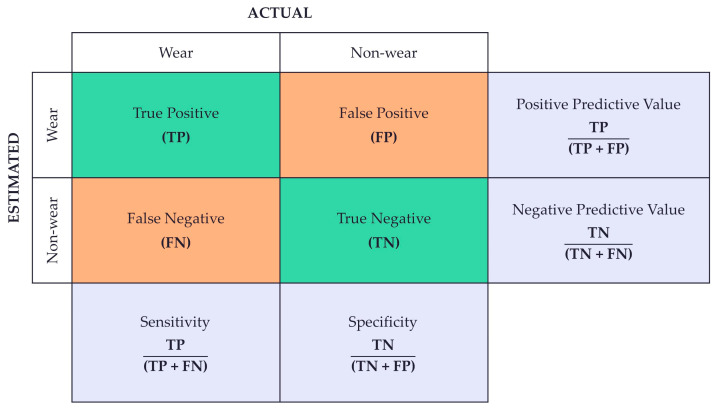
The equations used to calculate device accuracy as sensitivity, specificity, positive predictive value, and negative predictive value.

The accuracy of the device was reported as the ability to identify true positives correctly (sensitivity), the ability to identify true negatives correctly (specificity), the proportion of positive results that are true positives (positive predictive value), and the proportion of negative results that are true negatives (negative predictive value) (Figure 7). To assess the device’s reliability and level of agreement between logged and estimated wear time, the Cohen’s kappa was calculated and interpreted [47].

Paired comparisons to further evaluate the device’s accuracy were performed using paired *t*-tests (*p* < 0.05) after the differences between groups under comparison were checked to ensure they were normally distributed using the Shapiro–Wilk test (*p* > 0.05). Where differences were not normally distributed, the Wilcoxon signed-rank test (*p* > 0.05) was utilised instead. For complete results of the normality tests, please refer to the Supplementary Material. Tests were carried out to identify statistical differences between FSR and accelerometer-estimated wear time, the device’s accuracy at the original and lower sampling rates, and the accuracy of FSR and accelerometer-determined wear time estimations during each activity of the SHAP.

To explore the device’s accuracy at lower sampling rates, the dataset obtained from both the FSR and the accelerometer at a rate of 1 Hz were downsampled to reflect sampling rates of 1/2 Hz, 1/5 Hz, 1/10 Hz, 1/15 Hz, 1/20 Hz, 1/30 Hz, and 1/60 Hz, simulating the collection of compliance data every 2 s, 5 s, 10 s, 15 s, 20 s, 30 s, and a minute, respectively. Device accuracy was calculated as the percentage agreement between actual and sensor-based wear time estimations. The accuracy of the device for each compliance monitoring method (FSR and accelerometer) at each simulated sampling rate was compared to the device’s accuracy at the original sampling rate of 1 Hz. Specifically, for each simulated sampling rate, a paired comparison was conducted to check for statistical differences between the device’s accuracy at the original and simulated sampling rate.

The accuracy of FSR and accelerometer-determined wear time estimations during each activity of the SHAP was calculated for each participant and visualised. Participants wore their instrumented orthosis throughout the completion of the SHAP, and thus, any estimates claiming non-wear during this time, that is, data values that fell below the threshold, were classed as false negatives. It is important to note that some activities, such as rotating a key, are, by nature, short in duration and in some instances, only lasted a second. Entries from user feedback questionnaires were collated and visualised. Responses from users that were on a scale of 1 to 10 were averaged whilst written feedback were grouped into common themes that were summarised into keywords by the investigator and visualised. Data analyses and visualisations were carried out using MATLAB (version R2022b, The MathWorks, Inc., Natick, MA, USA), Microsoft Excel (version 2409, Microsoft Corporation, Redmond, WA, USA), and SPSS Statistics for Windows (version 26.0, IBM, Armonk, NY, USA).

## 3. Results

Fifteen healthy volunteers (28.7 ± 8.6 years; range: 22–53 years; 5 male, 10 female) participated in the feasibility testing of the device.

FSR-based wear time estimations were found to have a specificity of 92.7 ± 16.4% and sensitivity of 74.0 ± 41.3%, whilst accelerometer-based wear time estimates had a specificity of 66.1 ± 34.7% and sensitivity of 86.2 ± 8.0%. Moreover, FSR-based estimations had a positive predictive value of 97.3 ± 5.7% and a negative predictive value of 76.7 ± 32.4% whilst accelerometer-based estimates had a positive predictive value of 88.5 ± 11.8% and a negative predictive value of 60.9 ± 23.9%. Calculations of the Cohen’s kappa coefficient revealed a moderate level of agreement (0.66 ± 0.41) between actual and FSR-determined wear time and a weak level of agreement (0.49 ± 0.34) between actual and accelerometer-determined wear time.

**Figure 8:**
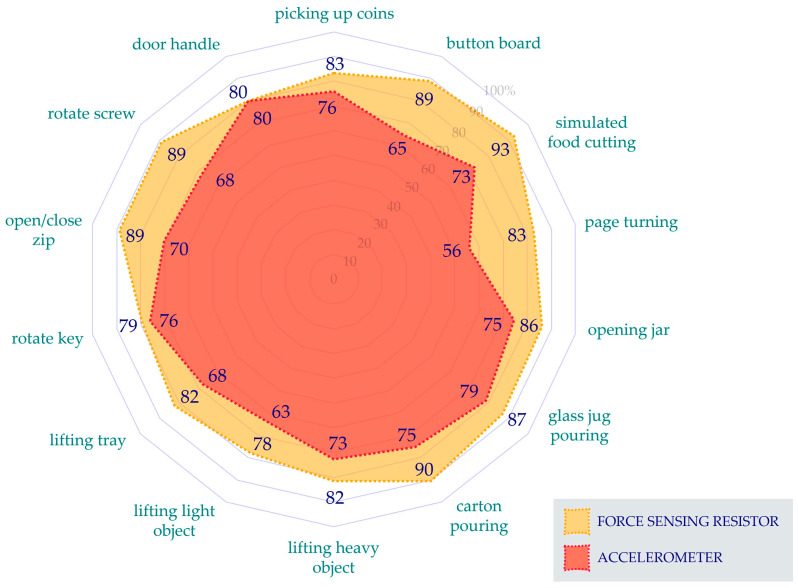
Average accuracy of wear time estimations, by the force sensing resistor (FSR) and accelerometer, whilst participants completed activities of daily living (ADLs) as part of the Southampton Hand Assessment Procedure (SHAP).

No difference was found between FSR and accelerometer-based wear time estimations (*p* = 0.733). Furthermore, no differences were found between the accuracies at the original sampling rate (1 Hz) and each simulated sampling rates for both FSR and accelerometer-determined wear time estimations (*p* > 0.133). During the first part of the SHAP, there were no differences in the accuracy of wear time estimations between FSR and accelerometer-derived estimates (*p* > 0.176). However, during the completion of the ADLs in the second part of the SHAP, FSR-based wear time estimations were more accurate than those obtained by the accelerometer during the button board activity (*p* = 0.000), simulated food cutting (*p* = 0.009), carton pouring (*p* = 0.019), and when rotating a screw (*p* = 0.006) (Figure 8).

**Figure 9:**
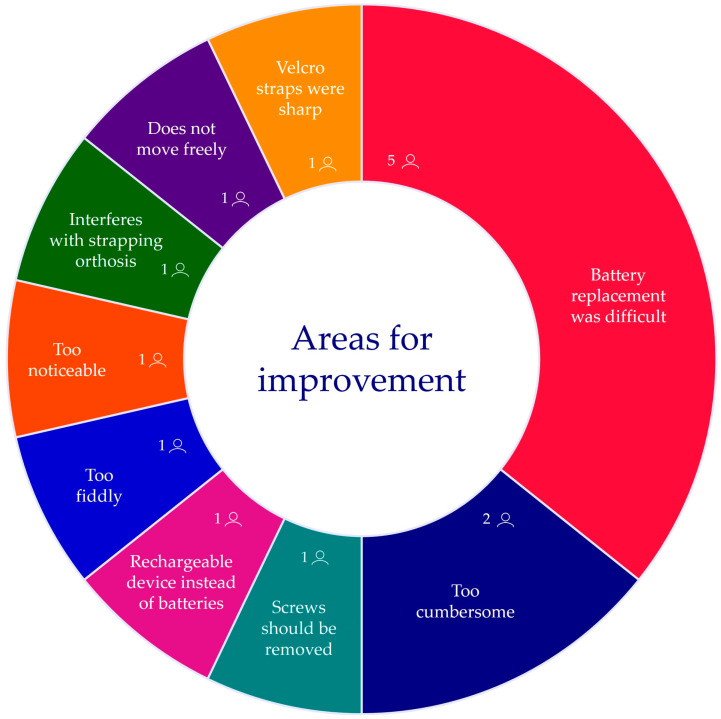
User feedback obtained regarding the issues they faced and thus, areas of improvement for the device. Feedback segment size is proportional to the frequency of said feedback received.

Feedback received on a scale of 1 (low) to 10 (high) revealed that users generally felt comfortable wearing the orthosis instrumented with the device during the protocol (7.54 ± 1.37) but believed it would be less comfortable to wear for longer periods (6.40 ± 1.64). The process of removing and replacing the battery was also the most frequent concern reported by users (Figure 9).

## 4. Discussion

Overall, the FSR was better at determining the times when the orthosis was not worn whilst the accelerometer-based wear time estimations were better at discerning wear time than non-wear. The FSR-determined wear time estimations were more reliable than those obtained from the accelerometer, and it can be inferred that when FSR-based wear time estimations recognise that the orthosis is worn, it is accurate. When compared to FSR-based estimations, non-wear time estimations from the accelerometer are less accurate overall, but due to having a higher sensitivity (86.2%) than the FSR (74.0%), accelerometer-determined wear time is usually more correct than that obtained from the FSR.

One reason for this performance could be the way in which these sensors function—the FSR relies on pressure directly applied to the sensor to determine wear time, whilst the accelerometer infers wear time from movement. As the device can still experience movement whilst the orthosis is not worn, the accelerometer-based estimations are less likely to determine non-wear time accurately [48]. At the same time, the FSR, requiring the application of pressure to estimate wear time, would estimate non-wear more accurately. However, when the orthosis is donned, the FSR can only accurately calculate wear-time if it experiences pressure throughout the duration of wear and consequently may underestimate wear time, should there be the absence of pressure applied on the FSR at any time.

Equally important to consider is the fact that accelerometer-based estimations are less dependent on the morphology of the orthosis in comparison to FSR-determined wear time estimations. Namely, due to the nature of the FSR, these wear time estimations are directly affected by the region of sensor placement within the orthosis, how the sensor is adhered to the orthosis, the surface curvature of the orthosis at the region of placement, how well the orthosis is fitted on the user, and the contact area at the FSR-skin interface. Furthermore, whilst the accelerometer is directly embedded on the miniature single board computer, the FSR is attached to the board externally using wires. Therefore, faulty connections to the board could compromise the accuracy of the data obtained from the FSR.

Comparisons between percentage agreement and sampling rate demonstrated that the accuracy did not vary when the sampling rate was reduced. However, data collected over a longer period are necessary to infer and justify the need to lower the sampling rate. Whilst a lower sampling rate would decrease the amount of data collected and therefore, the amount of phone storage taken and app data acquired, it should not compromise on the accuracy of wear time estimations, as it has been reported in previous studies collecting data at sampling rates lower than that used in this study, that it is possible to miss when the orthosis was donned or doffed [28,49].

User feedback revealed that some of the issues faced whilst wearing the orthosis instrumented with the compliance device, such as discomfort and skin irritation, were strikingly synonymous to reasons why patients decline to wear their prescribed orthoses as recommended [16,17,18]. Many patients have had concerns about the aesthetic value of orthoses [18,19,50] and the addition of a compliance monitoring device that increases the visibility of the orthosis may negatively impact compliance further. Thus, it is vital to ensure that the addition of a compliance monitoring device to an orthosis does not contribute to any discomfort, exhaustion, or low aesthetic value that may be caused by an orthosis.

Previous studies have employed microcontrollers like the Arduino Uno and have obtained readings from a computer after a period of data collection. However, these microcontrollers were not viable options due to their large form factor when compared to hand orthoses. This device, with a smaller form factor than previous compliance monitoring systems, is envisioned as a device that can be used alongside any orthosis, applied to any anatomical region. Although currently instrumented on the Push^®^ ortho Thumb Brace CMC, it is hypothesised that what would work for smaller, upper limb orthoses would also be applicable and usable for other orthosis types such as spinal, hip, and lower limb orthoses. However, it is important to acknowledge that orthoses are prescribed to treat a variety of conditions, and depending on the pathology in question, for example, joint deformities or burns, it may not be possible to achieve contact with the skin, inevitably impacting FSR-based wear time estimations. Nevertheless, the use of both the accelerometer and FSR to monitor compliance proves to be advantageous in this scenario, as the accelerometer will still be able to estimate wear time, even when there is no contact with the skin. Likewise, in situations where the orthotic user is sedentary or inactive but contact with the skin is possible, the use of the FSR alongside the accelerometer, will be valuable. Furthermore, all the materials used to prototype the device were off-the-shelf components which enables this system to be replicated in settings with minimal manufacturing resources.

With the simultaneous use of a pressure sensor and accelerometer to monitor compliance in hand orthoses, this study has described a novel technique to monitor orthosis use in the upper limb. Furthermore, as identified by a previous systematic review [26], only six studies have used pressure sensors to monitor orthosis compliance, and five out of these six studies were on orthoses applied to the lower limb [51,52,53,54,55]. The one study that used pressure sensors for upper limb orthoses used it alongside temperature sensors but was only tested on two healthy participants [30]. Similarly, six studies utilised accelerometers to monitor the use of orthoses applied to the hip [56] and lower limb [48,57,58,59,60]. Only one study has been found to use accelerometers, alongside temperature sensors, to measure compliance in the upper limb [61]. Pressure sensors and accelerometers are looked upon favourably in comparison to temperature sensors and step counters [26] and were the preferred options for this device. Whilst temperature sensors have been successfully applied to monitor compliance in a variety of orthoses, including those of the upper limb, high temperatures due to high pressures applied by the orthosis, or a warmer climate, could compromise the accuracy of temperature sensors. Furthermore, many of these sensors were integrated within the orthosis itself. Consequently, the structure of the orthosis may be changed, which could lead to changes in the functioning of the orthosis. In contrast, this device was designed to not interfere with the architecture of the orthosis, but to be attached to it without affecting its shape and function. At the same time, a pressure sensor also has scope to provide measurements regarding the fitting of orthotic devices by measuring tightness, the forces applied to a specific part of the joint by measuring the pressures at the orthosis-skin interface, and the variation of the forces applied to the affected area during different activities of daily living. In addition to this, the use of an accelerometer provides further information regarding the mobility of the user whilst they wear their orthosis, and this has the potential to give insights into user behaviour and the activities for which users are likely to wear their orthosis.

Essential to address are the limitations of this study. Whilst fifteen participants were recruited, they are not representative of the patients who would usually wear orthoses for hand pathology. Although this study was carried out to investigate the feasibility of a monitoring device, it may be possible that testing in a patient population would have given insights into how a patient would use their device and any problems they would face whilst using the compliance monitoring system alongside their orthosis. Furthermore, whilst the device was tested during various ADLs, the lack of rest periods whilst donning the orthosis highlights that lengthier device testing would be valuable to demonstrate the device’s true potential to monitor the daily use of orthotics.

## 5. Conclusions

In the past, studies have validated compliance monitoring systems using accelerometers [48,56,57,58,59] and pressure sensors [30,51,52,53,54,55]. Although the combination of an FSR and accelerometer has been used previously to monitor arm rehabilitation [62], using pressure sensors and accelerometers to monitor compliance and orthosis usage, simultaneously, is yet to be documented. This study has developed a wireless, portable orthosis compliance monitoring system that works alongside an app and does not modify the structure of the orthosis itself. The testing revealed that to provide accurate wear time estimations, FSR-based estimations require higher sensitivity whilst accelerometer-based estimations need higher specificity. Thus, FSR-determined wear time required better estimations whilst users donned their orthosis and accelerometer-based estimations needed to better detect when the orthosis was not worn. Whilst the results show that there are areas for improvement, this study has demonstrated the feasibility of using this compliance monitoring device and with a refined filtering algorithm, using data from both the FSR and accelerometer combined, this system has the potential to estimate wear time accurately and in real time.

## Data Availability

The original contributions presented in this study are included in the article/Supplementary Material. Further inquiries can be directed to the corresponding author.

## Supplementary Materials

The following supporting information can be downloaded at: https://www.mdpi.com/article/10.3390/s25051352/s1, Table S1: Participant demographics; Table S2: Force sensing resistor wear time estimations; Table S3: Force sensing resistor device accuracy; Table S4: Force sensing resistor percentage agreement vs. sampling rates; Table S5: Force sensing resistor percentage agreement during Southampton Hand Assessment Procedure (SHAP) – grip patterns; Table S6: Force sensing resistor percentage agreement during SHAP – activities of daily living; Table S7: Accelerometer wear time estimations; Table S8: Accelerometer device accuracy; Table S9: Accelerometer percentage agreement vs. sampling rates; Table S10: Accelerometer percentage agreement during Southampton Hand Assessment Procedure (SHAP) – grip patterns; Table S11: Accelerometer percentage agreement during SHAP – activities of daily living; Table S12: User feedback on a scale of 1 (low) to 10 (high); Table S13: Summary of written user feedback; Table S14: Statistical analyses: paired groups & Shapiro-Wilk test results; Table S15: Statistical analyses: paired *t*-test results; Table S16: Statistical analyses: Wilcoxon signed-rank test results.
