# Supplementary material for: A Compact Orthosis Compliance Monitoring Device Using Pressure Sensors and Accelerometers: Design and Proof-of-Concept Testing
Source: Sensors (Basel). 2025 Feb 22;25(5):1352. doi: 10.3390/s25051352 (PMC11902658; doi:10.3390/s25051352)
Supplement: Supplementary file 1 [file sensors-25-01352-s001.zip › sensors-3385701-supplementary.pdf]

SUPPLEMENTARY MATERIAL (DATA)

PARTICIPANT INFORMATION

TABLE S1: PARTICIPANT DEMOGRAPHICS

	1	2	3	4	5	6	7	8	9	10	11	12	13	14	15
**Age (years)**	23	53	25	24	29	24	43	27	30	27	24	32	22	22	25
**Gender**	M	F	F	F	M	M	F	F	F	F	M	F	M	F	F
**Hand dominance**	R	R	R	R	R	L	R	R	R	R	R	L	R	R	R
**Hand circumference (cm)**	21.1	18.7	17.5	18.5	19.0	17.8	16.5	18.5	18.3	18.5	20.4	20.0	21.5	17.0	17.5
**Push Brace size**	R2	R1	R1	R1	R1	L1	R1	R1	R1	R1	R2	L2	R2	R1	R1

FORCE SENSING RESISTOR (FSR)

TABLE S2: WEAR-TIME ESTIMATIONS

	Wear		Non-wear	
	total readings taken	readings correctly identified	total readings taken	readings correctly identified
**1**	1355	115	483	1723
**2**	1485	1487	478	476
**3**	1439	1455	484	468
**4**	1421	1465	457	413
**5**	1099	1101	453	451
**6**	1372	1601	628	399
**7**	1704	1723	503	484
**8**	1388	1389	494	493
**9**	1297	1300	506	503
**10**	1334	1296	539	577
**11**	1264	147	472	1589
**12**	1429	110	569	1888
**13**	1157	56	498	1599
**14**	1408	1375	481	514
**15**	1313	1314	549	548

TABLE S3: DEVICE ACCURACY

	True Positive	False Positive	False Negative	True Negative	Sensitivity (%)	Specificity (%)	Positive Predictive Value (%)	Negative Predictive Value (%)	Percentage Agreement (%)	Cohen's Kappa
**1**	115	0	1240	483	8.49	100.00	100.00	28.03	32.54	0.05
**2**	1485	2	0	476	100.00	99.58	99.87	100.00	99.90	1.00
**3**	1185	270	254	214	82.35	44.21	81.44	45.73	72.75	0.27
**4**	1418	47	3	410	99.79	89.72	96.79	99.27	97.34	0.93
**5**	1099	2	0	451	100.00	99.56	99.82	100.00	99.87	1.00
**6**	1372	229	0	399	100.00	63.54	85.70	100.00	88.55	0.71
**7**	1704	19	0	484	100.00	96.22	98.90	100.00	99.14	0.98
**8**	1388	1	0	493	100.00	99.80	99.93	100.00	99.95	1.00
**9**	1296	4	1	502	99.92	99.21	99.69	99.80	99.72	0.99
**10**	1296	0	38	539	97.15	100.00	100.00	93.41	97.97	0.95
**11**	146	1	1118	471	11.55	99.79	99.32	29.64	35.54	0.07
**12**	110	0	1319	569	7.70	100.00	100.00	30.14	33.98	0.05
**13**	55	1	1102	497	4.75	99.80	98.21	31.08	33.35	0.03
**14**	1374	1	34	480	97.59	99.79	99.93	93.39	98.15	0.95
**15**	1313	1	0	548	100.00	99.82	99.92	100.00	99.95	1.00

TABLE S4: PERCENTAGE AGREEMENT VS. SAMPLING RATES

	1 Hz	1/2 Hz	1/5 Hz	1/10 Hz	1/15 Hz	1/20 Hz	1/30 Hz	1/60 Hz
1	32.54	32.43	32.61	33.15	32.52	38.04	33.87	45.16
2	99.90	100.00	99.75	100.00	99.24	100.00	100.00	100.00
3	72.75	72.87	70.91	72.02	72.09	74.23	73.85	72.73
4	97.34	97.44	96.81	96.81	96.03	96.81	95.24	93.75
5	99.87	99.87	99.68	100.00	100.00	100.00	100.00	100.00
6	88.55	88.90	88.25	88.00	88.06	89.00	86.57	85.29
7	99.14	99.09	99.10	99.10	97.97	98.20	98.65	97.30
8	99.95	99.89	100.00	100.00	100.00	100.00	100.00	100.00
9	99.72	99.78	100.00	100.00	100.00	100.00	100.00	100.00
10	97.97	97.97	98.13	97.87	98.40	97.87	98.41	100.00
11	35.54	35.48	34.20	33.33	32.76	31.03	32.76	34.48
12	33.98	33.73	34.00	32.50	34.33	31.00	32.84	29.41
13	33.35	33.21	34.14	35.54	36.04	30.12	35.71	32.14
14	98.15	97.99	98.41	98.41	97.62	97.89	96.83	96.88
15	99.95	100.00	100.00	100.00	100.00	100.00	100.00	100.00

TABLE S5: PERCENTAGE AGREEMENT DURING SOUTHAMPTON HAND ASSESSMENT PROCEDURE (SHAP) – GRIP PATTERNS

	Light						Heavy					
	Spherical	Tripod	Power	Lateral	Tip	Extension	Spherical	Tripod	Power	Lateral	Tip	Extension
1	0.00	0.00	0.00	0.00	0.00	25.00	33.33	0.00	25.00	33.33	25.00	50.00
2	100	100	100	100	100	100	100	100	100	100	100	100
3	100.00	100.00	100.00	100.00	100.00	100.00	100.00	100.00	100.00	100.00	100.00	100.00
4	100.00	100.00	100.00	100.00	100.00	100.00	100.00	100.00	100.00	100.00	100.00	100.00
5	100.00	100.00	100.00	100.00	100.00	100.00	100.00	100.00	100.00	100.00	100.00	100.00
6	100.00	100.00	100.00	100.00	100.00	100.00	100.00	100.00	100.00	100.00	100.00	100.00
7	100.00	100.00	100.00	100.00	100.00	100.00	100.00	100.00	100.00	100.00	100.00	100.00
8	100.00	100.00	100.00	100.00	100.00	100.00	100.00	100.00	100.00	100.00	100.00	100.00
9	100.00	100.00	100.00	100.00	100.00	100.00	100.00	100.00	100.00	100.00	100.00	100.00
10	100.00	100.00	100.00	100.00	100.00	100.00	100.00	100.00	100.00	100.00	100.00	100.00
11	0.00	0.00	0.00	0.00	0.00	0.00	0.00	33.33	0.00	33.33	0.00	33.33
12	50.00	33.33	33.33	33.33	0.00	0.00	66.67	33.33	0.00	33.33	25.00	33.33
13	33.33	66.67	33.33	33.33	50.00	25.00	33.33	33.33	33.33	50.00	33.33	25.00
14	100.00	100.00	100.00	100.00	100.00	100.00	100.00	100.00	100.00	100.00	100.00	100.00
15	100.00	100.00	100.00	100.00	100.00	100.00	100.00	100.00	100.00	100.00	100.00	100.00

TABLE S6: PERCENTAGE AGREEMENT DURING SHAP – ACTIVITIES OF DAILY LIVING (ADLS)

	Picking up coins	Button board	Simulated food cutting	Page turning	Opening jar	Glass jug pouring	Carton pouring	Lifting heavy object	Lifting light object	Lifting tray	Rotate key	Open/ close zip	Rotate screw	Door handle
1	25.00	50.00	62.50	0.00	50.00	75.00	46.15	50.00	0.00	25.00	25.00	50.00	66.67	33.33
2	100	100	100	100	100	100	100	100	100	100	100	100	100	100
3	85.71	100.00	100.00	100.00	100.00	100.00	100.00	66.67	100.00	100.00	100.00	100.00	100.00	100.00
4	100.00	100.00	100.00	100.00	100.00	100.00	100.00	100.00	100.00	100.00	100.00	100.00	100.00	100.00
5	100.00	100.00	100.00	100.00	100.00	100.00	100.00	100.00	100.00	100.00	100.00	100.00	100.00	100.00
6	100.00	100.00	100.00	100.00	100.00	100.00	100.00	100.00	100.00	100.00	100.00	100.00	100.00	100.00
7	100.00	100.00	100.00	100.00	100.00	100.00	100.00	100.00	100.00	100.00	100.00	100.00	100.00	100.00
8	100.00	100.00	100.00	100.00	100.00	100.00	100.00	100.00	100.00	100.00	100.00	100.00	100.00	100.00
9	100.00	100.00	100.00	100.00	100.00	100.00	100.00	100.00	100.00	100.00	100.00	100.00	100.00	100.00
10	100.00	100.00	100.00	100.00	100.00	100.00	100.00	100.00	100.00	100.00	100.00	100.00	100.00	100.00
11	50.00	54.55	87.50	50.00	50.00	0.00	83.33	33.33	33.33	0.00	33.33	80.00	60.00	66.67
12	71.43	88.89	75.00	25.00	25.00	71.43	72.73	25.00	33.33	40.00	0.00	75.00	60.00	0.00
13	20.00	38.46	66.67	66.67	66.67	60.00	54.55	50.00	0.00	66.67	33.33	25.00	50.00	0.00
14	100.00	100.00	100.00	100.00	100.00	100.00	100.00	100.00	100.00	100.00	100.00	100.00	100.00	100.00
15	100.00	100.00	100.00	100.00	100.00	100.00	100.00	100.00	100.00	100.00	100.00	100.00	100.00	100.00

ACCELEROMETER

TABLE S7: WEAR-TIME ESTIMATIONS

	Wear		Non-wear	
	total readings taken	readings correctly identified	total readings taken	readings correctly identified
1	1098	803	394	689
2	1222	1079	394	537
3	1166	1074	390	482
4	1148	1149	368	367
5	886	774	367	479
6	1109	1459	508	158
7	1376	1525	407	258
8	1141	1182	405	364
9	1043	1169	407	281
10	1080	1041	436	475
11	1037	819	387	605
12	1153	1105	458	506
13	937	1075	404	266
14	1145	1320	392	217
15	1066	912	448	602

TABLE S8: DEVICE ACCURACY

	True Positive	False Positive	False Negative	True Negative	Sensitivity (%)	Specificity (%)	Positive Predictive Value (%)	Negative Predictive Value (%)	Percentage Agreement (%)	Cohen's Kappa
1	784	19	314	375	71.40	95.18	97.63	54.43	77.68	0.54
2	1067	12	155	382	87.32	96.95	98.89	71.14	89.67	0.75
3	990	84	176	306	84.91	78.46	92.18	63.49	83.29	0.59
4	921	228	227	140	80.23	38.04	80.16	38.15	69.99	0.18
5	769	5	117	362	86.79	98.64	99.35	75.57	90.26	0.78
6	989	470	120	38	89.18	7.48	67.79	24.05	63.51	-0.04
7	1251	274	125	133	90.92	32.68	82.03	51.55	77.62	0.27
8	1024	158	117	247	89.75	60.99	86.63	67.86	82.21	0.52
9	977	192	66	215	93.67	52.83	83.58	76.51	82.21	0.51
10	1031	10	49	426	95.46	97.71	99.04	89.68	96.11	0.91
11	802	17	235	370	77.34	95.61	97.92	61.16	82.30	0.62
12	1095	10	58	448	94.97	97.82	99.10	88.54	95.78	0.90
13	678	397	259	7	72.36	1.73	63.07	2.63	51.08	-0.29
14	1099	221	46	171	95.98	43.62	83.26	78.80	82.63	0.46
15	885	27	181	421	83.02	93.97	97.04	69.93	86.26	0.70

TABLE S9: PERCENTAGE AGREEMENT VS. SAMPLING RATES

	1 Hz	1/2 Hz	1/5 Hz	1/10 Hz	1/15 Hz	1/20 Hz	1/30 Hz	1/60 Hz
1	77.68	78.02	77.26	76.67	78.00	72.00	80.00	72.00
2	89.67	88.99	88.27	88.27	86.11	86.42	88.89	92.59
3	83.29	82.26	81.73	80.13	81.73	82.05	78.85	73.08
4	69.99	70.45	69.74	71.05	62.75	69.74	66.67	65.38
5	90.26	89.47	91.24	90.48	88.10	92.06	92.86	95.24
6	63.51	64.03	64.81	64.20	61.11	59.26	55.56	59.26
7	77.62	77.58	77.59	79.33	79.83	81.11	78.33	80.00
8	82.21	81.89	80.97	81.94	81.73	80.77	80.77	88.46
9	82.21	82.48	83.79	83.45	82.47	84.93	79.59	80.00
10	96.11	96.04	97.04	98.03	98.04	98.68	98.04	100.00
11	82.30	82.72	82.11	81.82	85.26	83.33	81.25	87.50
12	95.78	95.53	95.98	95.06	96.30	92.59	94.44	92.59
13	51.08	52.31	52.79	56.30	55.56	50.00	53.33	47.83
14	82.63	82.57	83.44	81.17	87.38	76.62	84.62	84.62
15	86.26	86.39	85.48	85.53	81.19	81.58	80.39	69.23

TABLE S10: PERCENTAGE AGREEMENT DURING SOUTHAMPTON HAND ASSESSMENT PROCEDURE (SHAP) – GRIP PATTERNS

	Light						Heavy					
	Spherical	Tripod	Power	Lateral	Tip	Extension	Spherical	Tripod	Power	Lateral	Tip	Extension
**1**	0.00	66.67	100.00	66.67	66.67	25.00	100.00	66.67	50.00	66.67	66.67	50.00
**2**	100.00	75.00	100.00	33.33	0.00	66.67	66.67	66.67	100.00	100.00	75.00	66.67
**3**	50.00	66.67	100.00	66.67	100.00	33.33	100.00	100.00	100.00	100.00	100.00	100.00
**4**	100.00	50.00	100.00	100.00	50.00	66.67	100.00	0.00	100.00	33.33	33.33	100.00
**5**	50.00	0.00	66.67	100.00	50.00	100.00	100.00	100.00	100.00	100.00	50.00	66.67
**6**	50.00	66.67	66.67	100.00	50.00	100.00	50.00	75.00	50.00	50.00	66.67	50.00
**7**	50.00	50.00	100.00	50.00	100.00	100.00	66.67	50.00	50.00	50.00	33.33	33.33
**8**	100.00	50.00	25.00	100.00	50.00	66.67	100.00	66.67	75.00	100.00	66.67	50.00
**9**	100.00	100.00	100.00	50.00	100.00	100.00	100.00	100.00	100.00	66.67	100.00	66.67
**10**	100.00	100.00	33.33	100.00	66.67	100.00	75.00	100.00	50.00	66.67	100.00	66.67
**11**	100.00	100.00	50.00	100.00	100.00	33.33	66.67	100.00	66.67	50.00	50.00	66.67
**12**	100.00	100.00	100.00	100.00	100.00	100.00	100.00	50.00	0.00	100.00	100.00	100.00
**13**	66.67	100.00	66.67	66.67	100.00	66.67	50.00	0.00	66.67	66.67	33.33	66.67
**14**	66.67	66.67	100.00	100.00	100.00	100.00	100.00	100.00	100.00	50.00	100.00	100.00
**15**	100.00	66.67	66.67	100.00	75.00	100.00	100.00	100.00	66.67	33.33	100.00	100.00

TABLE S11: PERCENTAGE AGREEMENT DURING SHAP – ACTIVITIES OF DAILY LIVING (ADLS)

	Picking up coins	Button board	Simulated food cutting	Page turning	Opening jar	Glass jug pouring	Carton pouring	Lifting heavy object	Lifting light object	Lifting tray	Rotate key	Open/ close zip	Rotate screw	Door handle
**1**	100.00	58.33	33.33	50.00	66.67	57.14	30.00	33.33	100.00	25.00	66.67	75.00	50.00	100.00
**2**	80.00	50.00	100.00	25.00	50.00	80.00	87.50	100.00	50.00	66.67	100.00	66.67	60.00	50.00
**3**	83.33	77.78	100.00	100.00	50.00	75.00	75.00	100.00	50.00	50.00	66.67	50.00	75.00	100.00
**4**	66.67	70.00	66.67	66.67	66.67	80.00	88.89	100.00	100.00	100.00	100.00	100.00	75.00	100.00
**5**	83.33	50.00	42.86	0.00	50.00	71.43	22.22	66.67	50.00	100.00	66.67	66.67	75.00	50.00
**6**	60.00	53.33	85.71	66.67	100.00	100.00	80.00	0.00	100.00	66.67	100.00	66.67	66.67	66.67
**7**	50.00	80.00	50.00	50.00	100.00	100.00	87.50	33.33	33.33	50.00	100.00	100.00	75.00	100.00
**8**	66.67	83.33	75.00	0.00	100.00	60.00	70.00	100.00	33.33	75.00	75.00	80.00	20.00	33.33
**9**	80.00	44.44	75.00	66.67	66.67	60.00	100.00	100.00	100.00	100.00	100.00	0.00	100.00	50.00
**10**	100.00	72.22	71.43	100.00	66.67	83.33	88.89	100.00	66.67	100.00	50.00	100.00	50.00	100.00
**11**	80.00	66.67	85.71	100.00	66.67	83.33	66.67	100.00	100.00	50.00	66.67	25.00	75.00	50.00
**12**	66.67	62.50	100.00	100.00	75.00	83.33	100.00	75.00	0.00	100.00	66.67	100.00	60.00	100.00
**13**	60.00	27.27	80.00	50.00	100.00	100.00	55.56	50.00	50.00	33.33	33.33	50.00	66.67	100.00
**14**	80.00	87.50	88.89	33.33	100.00	85.71	100.00	100.00	66.67	50.00	100.00	100.00	75.00	100.00
**15**	80.00	86.67	33.33	33.33	60.00	60.00	77.78	33.33	50.00	50.00	50.00	75.00	100.00	100.00

USER FEEDBACK

TABLE S12: USER FEEDBACK ON A SCALE OF 1 (LOW) TO 10 (HIGH)

	1	2	3	4	5	6	7	8	9	10	11	12	13	14	15
**Device comfort during laboratory protocol**	8.5	7	8	-	8	7	8	10	7	9	7	7	7	8	4
**Device comfort if worn for longer durations**	9	9	7	5	8	7	7	5	4	8	5	6	6	6	4
**Ease of battery removal and replacement**	7	7	6	8	6	7	9	8	5	10	7	8	4	6	4

TABLE S13: SUMMARY OF WRITTEN USER FEEDBACK

Suggested improvement to device	Number of times mentioned	Suggested improvement to device	Number of times mentioned
Battery replacement was difficult	5	Too cumbersome	2
Velcro straps were sharp	1	Too noticeable	1
Does not move freely	1	Too fiddly	1
Interferes with strapping the orthosis	1	Rechargeable device instead of batteries	1

STATISTICAL ANALYSES

TABLE S14: PAIRED GROUPS & SHAPIRO-WILK TEST RESULTS

(Shapiro-Wilk test was conducted to check if differences between groups were normally distributed prior to checking if differences were statistically different)

#	Paired groups		Shapiro – Wilk results		
	Group 1	Group 2	Statistic	df	Sig.
1	FSR-estimated wear time	Accelerometer-estimated wear time	0.761	15	0.001*
2	FSR accuracy at original sampling rate - 1 Hz	FSR accuracy at sampling rate of 1/2 Hz	0.957	15	0.643
3	FSR accuracy at original sampling rate - 1 Hz	FSR accuracy at sampling rate of 1/5 Hz	0.839	15	0.012*
4	FSR accuracy at original sampling rate - 1 Hz	FSR accuracy at sampling rate of 1/10 Hz	0.907	15	0.122
5	FSR accuracy at original sampling rate - 1 Hz	FSR accuracy at sampling rate of 1/15 Hz	0.899	15	0.092
6	FSR accuracy at original sampling rate - 1 Hz	FSR accuracy at sampling rate of 1/20 Hz	0.869	15	0.032*
7	FSR accuracy at original sampling rate - 1 Hz	FSR accuracy at sampling rate of 1/30 Hz	0.969	15	0.844
8	FSR accuracy at original sampling rate - 1 Hz	FSR accuracy at sampling rate of 1/60 Hz	0.704	15	0.000*
9	Accelerometer accuracy at original sampling rate - 1 Hz	Accelerometer accuracy at sampling rate of 1/2 Hz	0.973	15	0.904
10	Accelerometer accuracy at original sampling rate - 1 Hz	Accelerometer accuracy at sampling rate of 1/5 Hz	0.948	15	0.489
11	Accelerometer accuracy at original sampling rate - 1 Hz	Accelerometer accuracy at sampling rate of 1/10 Hz	0.933	15	0.299
12	Accelerometer accuracy at original sampling rate - 1 Hz	Accelerometer accuracy at sampling rate of 1/15 Hz	0.976	15	0.931
13	Accelerometer accuracy at original sampling rate - 1 Hz	Accelerometer accuracy at sampling rate of 1/20 Hz	0.945	15	0.451
14	Accelerometer accuracy at original sampling rate - 1 Hz	Accelerometer accuracy at sampling rate of 1/30 Hz	0.922	15	0.203
15	Accelerometer accuracy at original sampling rate - 1 Hz	Accelerometer accuracy at sampling rate of 1/60 Hz	0.913	15	0.151
16	FSR accuracy during SHAP (spherical - light)	Accelerometer accuracy during SHAP (spherical - light)	0.856	15	0.021*
17	FSR accuracy during SHAP (tripod - light)	Accelerometer accuracy during SHAP (tripod - light)	0.914	15	0.155
18	FSR accuracy during SHAP (power - light)	Accelerometer accuracy during SHAP (power - light)	0.941	15	0.394
19	FSR accuracy during SHAP (lateral - light)	Accelerometer accuracy during SHAP (lateral - light)	0.911	15	0.139
20	FSR accuracy during SHAP (tip - light)	Accelerometer accuracy during SHAP (tip - light)	0.919	15	0.186
21	FSR accuracy during SHAP (extension - light)	Accelerometer accuracy during SHAP (extension - light)	0.864	15	0.028*
22	FSR accuracy during SHAP (spherical - heavy)	Accelerometer accuracy during SHAP (spherical - heavy)	0.892	15	0.071
23	FSR accuracy during SHAP (tripod - heavy)	Accelerometer accuracy during SHAP (tripod - heavy)	0.914	15	0.154
24	FSR accuracy during SHAP (power - heavy)	Accelerometer accuracy during SHAP (power - heavy)	0.900	15	0.094
25	FSR accuracy during SHAP (lateral - heavy)	Accelerometer accuracy during SHAP (lateral - heavy)	0.934	15	0.317
26	FSR accuracy during SHAP (tip - heavy)	Accelerometer accuracy during SHAP (tip - heavy)	0.931	15	0.287
27	FSR accuracy during SHAP (extension - heavy)	Accelerometer accuracy during SHAP (extension - heavy)	0.933	15	0.302
28	FSR accuracy during SHAP (picking up coins)	Accelerometer accuracy during SHAP (picking up coins)	0.881	15	0.050
29	FSR accuracy during SHAP (button board)	Accelerometer accuracy during SHAP (button board)	0.946	15	0.462
30	FSR accuracy during SHAP (simulated food cutting)	Accelerometer accuracy during SHAP (simulated food cutting)	0.978	15	0.952
31	FSR accuracy during SHAP (page turning)	Accelerometer accuracy during SHAP (page turning)	0.941	15	0.390
32	FSR accuracy during SHAP (jar lid)	Accelerometer accuracy during SHAP (jar lid)	0.910	15	0.133
33	FSR accuracy during SHAP (glass jug pouring)	Accelerometer accuracy during SHAP (glass jug pouring)	0.816	15	0.006*
34	FSR accuracy during SHAP (carton pouring)	Accelerometer accuracy during SHAP (carton pouring)	0.853	15	0.019*
35	FSR accuracy during SHAP (lifting heavy object)	Accelerometer accuracy during SHAP (lifting heavy object)	0.918	15	0.178
36	FSR accuracy during SHAP (lifting light object)	Accelerometer accuracy during SHAP (lifting light object)	0.854	15	0.020*
37	FSR accuracy during SHAP (lifting tray)	Accelerometer accuracy during SHAP (lifting tray)	0.855	15	0.021*
38	FSR accuracy during SHAP (rotate key)	Accelerometer accuracy during SHAP (rotate key)	0.909	15	0.131
39	FSR accuracy during SHAP (open/close zip)	Accelerometer accuracy during SHAP (open/close zip)	0.925	15	0.226
40	FSR accuracy during SHAP (rotate screw)	Accelerometer accuracy during SHAP (rotate screw)	0.936	15	0.334
41	FSR accuracy during SHAP (door handle)	Accelerometer accuracy during SHAP (door handle)	0.857	15	0.022*

(* $p < 0.05$ = not normally distributed)

TABLE S15: PAIRED T-TEST RESULTS

(Paired t-tests were conducted to check for statistical differences between groups whose differences were normally distributed)

#	Paired groups		Paired t-test results					
	Group 1	Group 2	Mean	Standard deviation	t-statistic	df	p-value	Cohen's d
2	FSR accuracy at original sampling rate - 1 Hz	FSR accuracy at sampling rate of 1/2 Hz	0.003	0.145	0.089	14	0.930	0.023
4	FSR accuracy at original sampling rate - 1 Hz	FSR accuracy at sampling rate of 1/10 Hz	0.131	0.974	0.522	14	0.610	0.135
5	FSR accuracy at original sampling rate - 1 Hz	FSR accuracy at sampling rate of 1/15 Hz	0.243	1.164	0.807	14	0.433	0.208
7	FSR accuracy at original sampling rate - 1 Hz	FSR accuracy at sampling rate of 1/30 Hz	0.265	1.392	0.736	14	0.474	0.190
9	Accelerometer accuracy at original sampling rate - 1 Hz	Accelerometer accuracy at sampling rate of 1/2 Hz	-0.009	0.579	-0.058	14	0.955	-0.015
10	Accelerometer accuracy at original sampling rate - 1 Hz	Accelerometer accuracy at sampling rate of 1/5 Hz	-0.109	1.078	-0.393	14	0.700	-0.101
11	Accelerometer accuracy at original sampling rate - 1 Hz	Accelerometer accuracy at sampling rate of 1/10 Hz	-0.189	1.945	-0.376	14	0.713	-0.097
12	Accelerometer accuracy at original sampling rate - 1 Hz	Accelerometer accuracy at sampling rate of 1/15 Hz	0.336	3.408	0.382	14	0.708	0.099
13	Accelerometer accuracy at original sampling rate - 1 Hz	Accelerometer accuracy at sampling rate of 1/20 Hz	1.297	3.153	1.594	14	0.133	0.411
14	Accelerometer accuracy at original sampling rate - 1 Hz	Accelerometer accuracy at sampling rate of 1/30 Hz	1.134	3.240	1.356	14	0.197	0.350
15	Accelerometer accuracy at original sampling rate - 1 Hz	Accelerometer accuracy at sampling rate of 1/60 Hz	1.521	6.442	0.915	14	0.376	0.236
17	FSR accuracy during SHAP (tripod - light)	Accelerometer accuracy during SHAP (tripod - light)	9.443	54.269	0.674	14	0.511	0.174
18	FSR accuracy during SHAP (power - light)	Accelerometer accuracy during SHAP (power - light)	-0.557	47.400	-0.045	14	0.964	-0.012
19	FSR accuracy during SHAP (lateral - light)	Accelerometer accuracy during SHAP (lateral - light)	-4.446	46.491	-0.370	14	0.717	-0.096
20	FSR accuracy during SHAP (tip - light)	Accelerometer accuracy during SHAP (tip - light)	2.777	58.898	0.183	14	0.858	0.047
22	FSR accuracy during SHAP (spherical - heavy)	Accelerometer accuracy during SHAP (spherical - heavy)	-2.778	33.284	-0.323	14	0.751	-0.083
23	FSR accuracy during SHAP (tripod - heavy)	Accelerometer accuracy during SHAP (tripod - heavy)	8.332	41.905	0.770	14	0.454	0.199
24	FSR accuracy during SHAP (power - heavy)	Accelerometer accuracy during SHAP (power - heavy)	5.555	32.683	0.658	14	0.521	0.170
25	FSR accuracy during SHAP (lateral - heavy)	Accelerometer accuracy during SHAP (lateral - heavy)	14.443	39.274	1.424	14	0.176	0.368
26	FSR accuracy during SHAP (tip - heavy)	Accelerometer accuracy during SHAP (tip - heavy)	7.222	40.809	0.685	14	0.504	0.177
27	FSR accuracy during SHAP (extension - heavy)	Accelerometer accuracy during SHAP (extension - heavy)	10.554	37.331	1.095	14	0.292	0.283
28	FSR accuracy during SHAP (picking up coins)	Accelerometer accuracy during SHAP (picking up coins)	7.698	33.235	0.897	14	0.385	0.232
29	FSR accuracy during SHAP (button board)	Accelerometer accuracy during SHAP (button board)	24.124	20.267	4.610	14	0.000*	1.190
30	FSR accuracy during SHAP (simulated food cutting)	Accelerometer accuracy during SHAP (simulated food cutting)	20.249	25.664	3.056	14	0.009*	0.789
31	FSR accuracy during SHAP (page turning)	Accelerometer accuracy during SHAP (page turning)	26.667	53.841	1.918	14	0.076	0.495
32	FSR accuracy during SHAP (jar lid)	Accelerometer accuracy during SHAP (jar lid)	11.555	32.290	1.386	14	0.187	0.358
35	FSR accuracy during SHAP (lifting heavy object)	Accelerometer accuracy during SHAP (lifting heavy object)	8.889	44.037	0.782	14	0.447	0.202
38	FSR accuracy during SHAP (rotate key)	Accelerometer accuracy during SHAP (rotate key)	3.332	32.703	0.395	14	0.699	0.102
39	FSR accuracy during SHAP (open/close zip)	Accelerometer accuracy during SHAP (open/close zip)	18.333	34.555	2.055	14	0.059	0.531
40	FSR accuracy during SHAP (rotate screw)	Accelerometer accuracy during SHAP (rotate screw)	20.889	25.315	3.196	14	0.006*	0.825

(* $p < 0.05$ = statistically different)

TABLE S16: WILCOXON SIGNED-RANK TEST RESULTS

(Wilcoxon signed-rank tests were conducted to check for statistical differences between groups whose differences were not normally distributed)

#	Paired groups		Wilcoxon signed-rank test results	
	Group 1	Group 2	Z-value	p-value
**1**	FSR-estimated wear time	Accelerometer-estimated wear time	-0.341	0.733
**3**	FSR accuracy at original sampling rate - 1 Hz	FSR accuracy at sampling rate of 1/5 Hz	-0.454	0.649
**6**	FSR accuracy at original sampling rate - 1 Hz	FSR accuracy at sampling rate of 1/20 Hz	-0.426	0.670
**8**	FSR accuracy at original sampling rate - 1 Hz	FSR accuracy at sampling rate of 1/60 Hz	-0.795	0.426
**16**	FSR accuracy during SHAP (spherical - light)	Accelerometer accuracy during SHAP (spherical - light)	-0.431	0.667
**21**	FSR accuracy during SHAP (extension - light)	Accelerometer accuracy during SHAP (extension - light)	0.000	1.000
**33**	FSR accuracy during SHAP (glass jug pouring)	Accelerometer accuracy during SHAP (glass jug pouring)	-1.473	0.141
**34**	FSR accuracy during SHAP (carton pouring)	Accelerometer accuracy during SHAP (carton pouring)	-2.343	0.019*
**36**	FSR accuracy during SHAP (lifting light object)	Accelerometer accuracy during SHAP (lifting light object)	-0.872	0.383
**37**	FSR accuracy during SHAP (lifting tray)	Accelerometer accuracy during SHAP (lifting tray)	-1.084	0.278
**41**	FSR accuracy during SHAP (door handle)	Accelerometer accuracy during SHAP (door handle)	-0.119	0.905

(* $p < 0.05$ = statistically different)
